# Chronic Intermittent Hypoxia Induces Early-Stage Metabolic Dysfunction Independently of Adipose Tissue Deregulation

**DOI:** 10.3390/antiox10081233

**Published:** 2021-07-30

**Authors:** Fátima O. Martins, Joana F. Sacramento, Elena Olea, Bernardete F. Melo, Jesus Prieto-Lloret, Ana Obeso, Asuncion Rocher, Paulo Matafome, Emilia C. Monteiro, Silvia V. Conde

**Affiliations:** 1 CEDOC, NOVA Medical School, Faculdade de Ciências Médicas, Universidade NOVA de Lisboa, 1150-082 Lisboa, Portugal; Fatima.martins@nms.unl.pt (F.O.M.); joana.sacramento@nms.unl.pt (J.F.S.); bernardete.melo@nms.unl.pt (B.F.M.); emilia.monteiro@nms.unl.pt (E.C.M.); 2 Departamento de Enfermeria, Universidad de Valladolid, 47005 Valladolid, Spain; elena.olea@uva.es; 3 Instituto de Biologia y Genetica Molecular (IBGM), Consejo Superior de Investigaciones Científicas, Universidad de Valladolid, 47005 Valladolid, Spain; jesus.prieto@uva.es (J.P.-L.); aobeso@ibgm.uva.es (A.O.); rocher@ibgm.uva.es (A.R.); 4 Departamento de Bioquimica, Biologia Molecular y Fisiologia, Universidad de Valladolid, 47005 Valladolid, Spain; 5 Institute of Clinical and Biomedical Research (iCBR), Faculty of Medicine, University of Coimbra, 3000-548 Coimbra, Portugal; paulo.matafome@uc.pt; 6 Instituto Politécnico de Coimbra, Coimbra Health School, 3045-093 Coimbra, Portugal

**Keywords:** obstructive sleep apnea, metabolic dysfunction, insulin resistance, adipose tissue, hypoxia, inflammation, oxidative stress

## Abstract

Several studies demonstrated a link between obstructive sleep apnea (OSA) and the development of insulin resistance. However, the main event triggering insulin resistance in OSA remains to be clarified. Herein, we investigated the effect of mild and severe chronic intermittent hypoxia (CIH) on whole-body metabolic deregulation and visceral adipose tissue dysfunction. Moreover, we studied the contribution of obesity to CIH-induced dysmetabolic states. Experiments were performed in male Wistar rats submitted to a control and high-fat (HF) diet. Two CIH protocols were tested: A mild CIH paradigm (5/6 hypoxic (5% O_2_) cycles/h, 10.5 h/day) during 35 days and a severe CIH paradigm (30 hypoxic (5% O_2_) cycles, 8 h/day) during 15 days. Fasting glycemia, insulinemia, insulin sensitivity, weight, and fat mass were assessed. Adipose tissue hypoxia, inflammation, angiogenesis, oxidative stress, and metabolism were investigated. Mild and severe CIH increased insulin levels and induced whole-body insulin resistance in control animals, effects not associated with weight gain. In control animals, CIH did not modify adipocytes perimeter as well as adipose tissue hypoxia, angiogenesis, inflammation or oxidative stress. In HF animals, severe CIH attenuated the increase in adipocytes perimeter, adipose tissue hypoxia, angiogenesis, and dysmetabolism. In conclusion, adipose tissue dysfunction is not the main trigger for initial dysmetabolism in CIH. CIH in an early stage might have a protective role against the deleterious effects of HF diet on adipose tissue metabolism.

## 1. Introduction

Obstructive sleep apnea (OSA), the most common sleep disorder, is characterized by repetitive episodes of airflow cessation (apnea) or airflow reduction (hypopnea) caused by an obstructed or collapsed upper airway during sleep, which results in intermittent hypoxemia and hypercapnia. It is estimated that 1 billion adults aged 30–69 years worldwide could have OSA [1]. Over the years, several studies have described the relationship between OSA and metabolic diseases. Intermittent hypoxia and sleep fragmentation lead to insulin resistance, type 2 diabetes, metabolic syndrome, and aggravates obesity [2]. This relationship is not unidirectional since obesity is considered a major risk factor for the development and progression of OSA [3,4]. In fact, the prevalence of OSA in people with metabolic syndrome is almost 70% [3,5] and with type 2 diabetes almost 50% [6,7]. However, high glycemia, insulin resistance, and hyperinsulinemia are also metabolic pathological features present in lean OSA patients [8,9], meaning that OSA may increase the risk of dysmetabolism independently of the traditional cardiometabolic risk factors, such as obesity. In agreement, animal data show that rats and mice exposed to different chronic intermittent hypoxia (CIH) paradigms exhibit pathological metabolic characteristics as insulin-resistance and glucose intolerance, in the absence of obesity [10,11,12,13]. In fact, CIH-induced weight loss, at least in mice, [10,13,14] and dependently on CIH severity [14].

Several mechanisms were proposed to explain the presence of dysmetabolic features in OSA: Increased sympathetic activation [15,16], deregulation of the hypothalamus-pituitary axis [17], generation of reactive oxygen species (ROS) [2], alteration in adipokines levels [11,18], alterations in gut microbiota [19,20,21,22,23], and inflammation of the adipose tissue [18,24,25,26]. More recently, the hypothesis that dysmetabolic states in OSA are due to adipose tissue inflammation has gained huge attention [27], as some evidences showed that CIH promotes alterations in insulin signaling pathways, a pro-inflammatory phenotype and polarizes THP1 cells into M1 pro-inflammatory macrophages in visceral adipose tissue [25]. However, there is still a lot of controversy if adipose tissue dysfunction is the main trigger on metabolic disruption in OSA, as well as on the mechanisms promoting this organ dysfunction [12,25,28,29].

Herein, we investigated the effect of mild and severe CIH on whole-body metabolic deregulation and on visceral adipose tissue dysfunction. Additionally, we studied the contribution of obesity to CIH-induced dysmetabolic states and to tissue dysfunction. Particularly, we focused on hypoxia, angiogenesis, inflammation, oxidative stress, and metabolic function of the adipose tissue. We provided mechanistic insights showing that although both mild and severe CIH prompted whole-body insulin resistance and hyperinsulinemia, these early dysmetabolic states are not associated with increased weight gain, adipocytes perimeter, adipose tissue hypoxia, and inflammation or oxidative stress. Moreover, we showed that obesity-induced whole body and adipose tissue dysfunction is associated with increased hypoxia, decreased angiogenesis together with increased oxidative stress in the adipose tissue, and alterations in its metabolism, without affecting inflammatory markers. Interestingly, CIH in obesity attenuated obesity-induced adipose tissue dysfunction.

## 2. Materials and Methods

### 2.1. Animals

Experiments were performed in male Wistar rats (250–350 g), aged 3 months obtained from the vivarium of the NOVA Medical School|Faculdade de Ciências Médicas of the Universidade NOVA de Lisboa, Lisboa, Portugal and from the vivarium of the Faculty of Medicine of the University of Valladolid, Spain. After randomization, animals were submitted to two different diets: A standard diet—control groups—(7.4% lipid and 75% carbohydrates, of which 4% were sugars and 17% protein; RM3, SDS—Special Diet Services, UK) or a high-fat (HF) diet (60% energy from fat; 61.6% fat + 20.3% carbohydrate + 19.1% protein; Test Diets, MS, USA). Animals were exposed to the diets during 35 days or 12 weeks, depending on the CIH protocol used. Animals were kept under temperature and humidity control (21 ± 1 °C; 55 ± 10% humidity) and a regular light (08.00–20.00 h) and dark (20.00–08.00 h) cycle, with food and water ad libitum. Body weight, energy, and liquid intake were monitored two times per week. At the end of the experiments, the rats were sacrificed by an intracardiac overdose of pentobarbital, except when heart puncture was performed to collect blood. Visceral adipose tissue was collected and frozen in liquid nitrogen or placed in 4% PFA for further analysis. Laboratory care was in accordance with the European Union Directive for Protection of Vertebrates Used for Experimental and Other Scientific Ends (2010/63/EU). Experimental protocols were approved by the NOVA Medical School, Faculdade de Ciências Médicas and the Valladolid University Ethics Committee.

### 2.2. Chronic Intermittent Hypoxia Protocols

**Figure 1:**
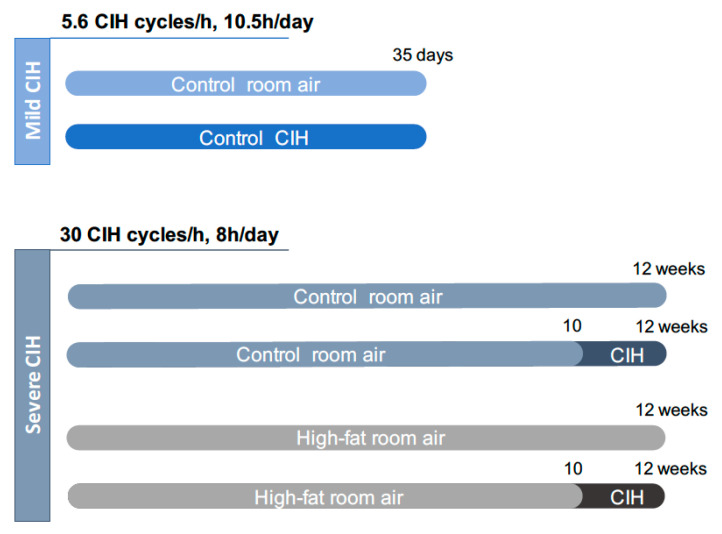
Experimental design of the study. The mild chronic intermittent hypoxia (CIH) paradigm consisted of 5.6 CIH cycles/h, 10.5 h/day, and it was performed in control animals (2.56 Kcal/g) during 35 days. The severe CIH paradigm consisted of 30 CIH cycles/h, 8 h/day, and it was performed in control and high-fat animals (5.1 Kcal/g) in the last 14 days of the diets, from weeks 10 to 12. Age-matched control or high-fat animals were exposed to circulating room air (21% O_2_ and 79% N_2_), in the same room and during the same time as the CIH animals to experience similar conditions.

Two paradigms of CIH were used: A mild CIH paradigm of 5.6 CIH cycles/h and a severe CIH paradigm of 30 CIH cycles/h (Figure 1). For the mild CIH paradigm, animals were housed in polypropylene cages, one per cage, as previously described by Sacramento et al. [12]. The cages were kept in a eucapnic atmosphere, inside of medium A-chambers (76 × 51 × 51 cm, A-60274-P, Biospherix Ltd., New York, NY, USA) and animals had ad libitum access to food and water. To guarantee the accuracy of CIH cycles, the chambers have gas injectors and sensors for oxygen (O_2_) and carbon dioxide (CO_2_) levels. The CO_2_ accumulation was avoided by the continuous flow of the gas mixtures, circulation of the gases inside the chambers through vent holes, and using self-indicating soda lime inside the chamber (AnalaR Normapur^®^ VWR International BVBA, Leuven, Belgium) to absorb the expired CO_2_. Inside the chamber, the CO_2_ levels never exceeded 1%. To control the oxygen levels inside the chamber, 100% nitrogen (N_2_) and 100% O_2_ were used via electronically regulated solenoid switches in a three-channel gas mixer. To achieve the mild CIH protocol, the oxygen levels in the chamber lowered gradually from 21 to 5% O_2_ (OxyCycler AT series, Biospherix Ltd., New York, NY, USA). To reduce the O_2_ levels to 5%, the chambers were infused with 100% N_2_ for 3.5 min and then were infused with 100% O_2_ for 7 min to restore O_2_ to ambient concentration of 21%, until the beginning of the next CIH cycle. Each cycle of mild CIH lasted 10.5 min (normoxic period: 3.5 min; hypoxic period: 7 min) and the animals were exposed during their sleep period (light phase of light/dark cycle) to 5.6 CIH cycles/h, 10.5 h/day (9:30 am to 8:00 pm), for 35 days. Throughout the remaining hours, the chambers were ventilated with a constant flow of room air to keep oxygen levels at 21%.

For the severe CIH paradigm, animals were housed hermetically shielding transparent methacrylate chambers (16 L, a maximum of four rats per chamber), with ad libitum access to food and water, as previously described by Gonzalez-Martín et al. [30]. Each chamber has a gas islet in front and inside the chamber, 3 cm away of the front wall of the chamber, there is an incomplete wall that breaks the gas jet smoothing and allows slowing the gas flow in the rats’ room. In the back wall of the chamber there are two outlets for the exit of the gas. In one of them, there is an O_2_ meter that allows monitoring the gas flowing out of the chamber continuously. The flow of gases into the chamber is controlled by electrovalves that are controlled by a microprocessor and allows determining the time and duration of entry of the desired gas. The animals were exposed to the severe CIH protocol that consisted of 30 cycles/h meaning cycles of exposure for 40 s to 5% O_2_, followed exposure for 80 s to air for 8 h per day, the equivalent to an apnoea-hypopnoea index of 30, for the last 14 days of the diets.

The mild CIH protocol was performed in control animals and the severe CIH protocol was performed in control and HF animals (Figure 1). Age-matched control or HF animals were exposed to room air (21% O_2_ and 79% N_2_), through the daylight and lights-off period, in the same room and during the same time as the CIH animals to experience similar conditions.

### 2.3. Measurement of Glycemia

At the end of the CIH protocols, fasting glycemia levels were evaluated in conscious animals by tail tipping after an overnight fast (approximately 16 h). Glucose levels were measured with a glucometer (Precision Xtra Meter, Abbott Diabetes Care, Amadora Portugal) and test strips (Abbott Diabetes Care, Amadora, Portugal).

### 2.4. Measurement of Insulin, Insulin Sensitivity, and Serum Non-Esterified Free Fatty Acids

Plasma was collected after heart puncture to ethylenediaminetetraacetic acid (EDTA) precoated tubes and were centrifuged (Sigma-Aldrich, Madrid, Spain) at 3000× *g* for 10 min (4 °C). The plasma and serum were stored at −80 °C in an ultralow freezer (Heraeus, Madrid, Spain). Insulin was quantified with an enzyme-linked immunosorbent assay (ELISA) kit (Mercodia Ultra-sensitive Rat Insulin ELISA Kit, Mercodia AB, Uppsala, Sweden). Insulin sensitivity was calculated with the homeostasis model assessment (HOMA-IR) index.

Non-esterified free fatty acids (NEFA) in the plasma were quantified using an in vitro enzymatic colorimetric method assay (Fujifilm Wako Chemicals GmbH, NEFA HR(2) kit, Neuss, Germany).

### 2.5. Western Blot Analysis

Visceral adipose tissue was homogenized in Zurich medium containing a cocktail of protease inhibitors [31]. Samples containing subcellular suspension were subjected to SDS-PAGE (10%) analysis and transferred onto nitrocellulose membrane (0.2 mmol/L; BioRad, Madrid, Spain) as described [31]. After blocking for 1 h at room temperature with 5% nonfat milk in Tris-buffered saline, pH 7.4 containing 0.1% Tween 20 (TTBS) (BioRad, Madrid, Spain), the membranes were incubated overnight at 4 °C with primary antibodies against interleukin 6 receptor (IL6 R, 1:100, Santa Cruz Biotechnology, Madrid, Spain), tumor necrosis factor alfa receptor (TNF-αR, 1:200, Santa Cruz Biotechnology, Madrid, Spain), interleukin 10 (1:200, SICGEN, Cantanhede, Portugal), F4/80 (1:1000, Abcam, Cambridge, UK), catalase (1:500, SICGEN, Cantanhede, Portugal), superoxide dismutase 1 (SOD1, 1:200, Santa Cruz Biotechnology, Madrid, Spain), P22Phox (1:200, Santa Cruz Biotechnology, Madrid, Spain), inducible nitric oxide synthase (iNOS, 1:200, Santa Cruz Biotechnology, Madrid, Spain), nuclear factor k B (NFkB, 1:200, Cell Signaling Technologies, Madrid, Spain), light polypeptide of nuclear factor k B alpha (IkBɑ, 1:200, Cell Signaling Technologies, Madrid, Spain), perilipin A (1:200, Cell Signaling Technologies, Madrid, Spain), peroxisome proliferator-activated receptor gamma (PPARƔ, 1:200, Cell Signaling Technologies, Madrid, Spain), glucose transporter type 4 (GLUT4; 1:200, Abcam, Cambridge, UK), and insulin receptor (IR; 1:500, Santa Cruz Biotechnology, Madrid, Spain). After three washing periods with TTBS, the membranes were incubated with anti-mouse (1:2000, Santa Cruz Biotechnology, Heidelberg, Germany) or anti-rabbit (1:2000, Santa Cruz Biotechnology, Heidelberg, Germany) or anti-goat (1:2000, Santa Cruz Biotechnology, Heidelberg, Germany) in TTBS for 1 h at room temperature and developed with enhanced chemiluminescence reagents according to the manufacturer’s instructions (Clarity Western ECL Substrate, BioRad, Madrid, Spain). The intensity of the signals was detected in a Chemidoc Molecular Imager (Chemidoc, BioRad, Madrid, Spain) and quantified using the Image Lab software (Bio-Rad, Madrid, Spain). The membranes were re-probed and tested to calnexin (1:1000, SICGEN, Cantanhede, Portugal) to compare and normalize the protein expression with the amount of protein loaded. The mean intensity of control samples in each membrane was used as a reference for controlling gel-to-gel variation.

### 2.6. Histochemical Analysis of the Adipose Tissue

Visceral adipose tissue samples previously collected and immersed-fixed in PFA 4% were embedded into paraffin (Sakura Finetek Europe B.V., Zoeterwoude, The Netherlands) and longitudinal serial sections of 10 μm thick were obtained with a Microtome Microm HM200 (MICROM Laborgeräte GmbH, ZEISS Group, Walldorf, Germany). After sectioning, the samples were transferred into slides and stained with hematoxylin and eosin. The perimeter of the adipocytes was measured using the software Fiji app for Image J (https://imagej.nih.gov/ij/, accessed From March to June 2021).

### 2.7. Immunohistochemical Analysis

After removing paraffin from the slides, the sections of the visceral adipose tissue were washed in PBS at room temperature (22–23 °C) for 5 min and incubated in a permeabilizing-blocking solution (PBS containing 0.1% Triton X-100 and 2% non-immunized goat serum) for 15 min, as previously described by Sacramento et al. [32]. The incubation with the primary antibodies, goat anti-hypoxia-inducible factor 1α (HIF-1α, 1:100; Sicgen, Cantanhede, Portugal), mouse anti-hypoxia-inducible factor 2α (HIF-2α, 1:100, Abcam, Cambridge, UK), goat anti-vascular endothelial growth factor (VEGF, 1:100, Sicgen, Cantanhede, Portugal) or goat anti-cluster of differentiation 31 (CD31, 1:100, Sicgen, Cantanhede, Portugal) was performed at 4 °C overnight. After washing with PBS (3 × 10 min), the sections were incubated with the secondary antibody (donkey anti-goat Alexa Fluor 546 or goat anti-mouse Alexa Fluor 550; Abcam, Cambridge, UK) at 1:5000 (for HIF-1α and HIF-2α) or 1:1000 (for VEGF and CD31) in a permeabilizing-blocking solution for 1 h at room temperature. The sections were washed with PBS (3 × 10 min) and were incubated with 4′,6-diamidino-2-phenylindole (DAPI) (1 μg/mL; Santa Cruz Biotechnology) for 5 min at room temperature. After washing with PBS (4 × 5 min), and with distilled water, the sections were mounted with Vectashield (Vector Laboratories, Burlingame, CA, USA). Negative controls were obtained by incubating the sections in the absence of primary antibody. Finally, the sections were examined with a fluorescence microscope (ZEISS Axio Imager 2; ZEISS, Oberkochen, Germany) with the excitation and emission filters for Alexa Fluor 546, Alexa Fluor 550 or DAPI. Finally, an Axiocam 105 colour (ZEISS) camera was used to capture the images of the sections. The Fiji app for Image J (https://imagej.nih.gov/ij/, accessed from March to June 2021) software allows measuring HIF-1ɑ, HIF-2ɑ, VEGF or CD31 fluorescence intensity in each section, that was used to calculate the relative intensity for the area per number of nucleus calculated by DAPI staining.

### 2.8. Statistical Analysis

Data were evaluated using the GraphPad Prism Software, version 6 (GraphPad Software Inc., San Diego, CA, USA) and showed mean values with SEM. The significance of the differences between the mean values was calculated by one- and two-way ANOVA with Bonferroni multiple comparison tests. Differences were considered significant at *p* < 0.05.

## 3. Results

### 3.1. Effect of Mild and Severe CIH on Insulin Sensitivity and Fasting Glycemia and Insulinemia

Table 1 depicts the effect of mild and severe CIH on fasting glycemia, fasting insulinemia, and insulin sensitivity in control animals. Exposure of the animals to mild and severe CIH did not modify fasting glycemia. As expected and as previously described [11,12], both mild and severe CIH protocols increased fasting insulin levels by 129 and 90%, respectively. Additionally, insulin sensitivity, given by the HOMA-IR, decreased in the animals exposed to both CIH paradigms, since the HOMA-IR values increased by 130 and 94% with mild and severe CIH, respectively (Table 1). Knowing that obesity is usually associated with CIH, and that sometimes the pathophysiological mechanisms behind the development of metabolic dysfunction in obesity and CIH might be difficult to differentiate, we evaluate the effect of obesity plus severe CIH. Obesity as expected did not modify fasting glycemia, but increased plasma insulin levels by 119% and induced insulin resistance, as observed by an increase of 148% in HOMA-IR (Table 1). Interestingly, severe CIH plus obesity did not change any of these metabolic parameters, since fasting glucose and insulin levels, as wells as insulin resistance were not modified when the HF animals were exposed during 14 days to severe CIH (Table 1).

### 3.2. Effect of CIH on Weight, Weight Gain, Retroperitoneal Fat, and Adipocytes Perimeter

**Figure 2:**
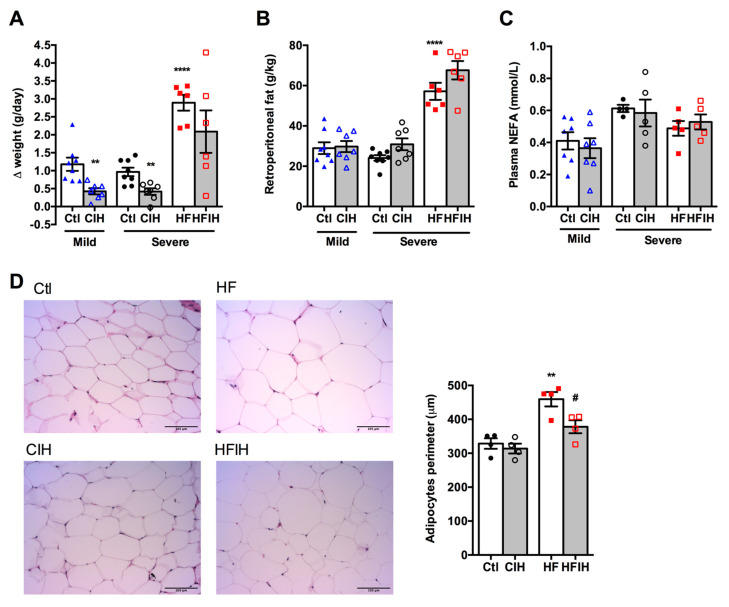
Effect of chronic intermittent hypoxia (CIH/IH) on weight gain, retroperitoneal fat, plasma non-esterified fatty acids (NEFA), and adipocytes perimeter. (**A**,**B**) Effect of mild and severe CIH on weight gain and retroperitoneal fat in control and high-fat (HF) animals, respectively. (**C**) Effect of mild and severe CIH exposure on NEFA in control and HF animals. (**D**) Effect of severe CIH on adipocytes perimeter in control and HF animals. Panels represent haematoxylin and eosin staining of adipose tissue in control, severe CIH, obese, and obese plus severe CIH animals. Animals were submitted to the mild CIH (5.6 CIH cycles/h, 10.5 h/day) during 35 days, while in the severe CIH (30 CIH cycles/h, 8 h/day). The control and HF animals were exposed to the protocol in the last 2 weeks of the diets. Data are presented as means ± SEM of 4–8 animals. One- and two-way ANOVA with Dunnett’s and Bonferroni multiple comparison tests, respectively: ** *p* < 0.01 and **** *p* < 0.0001 compared with control animals of the respective; # *p* < 0.05 compared values with HF animals without CIH.

Figure 2A,B shows the effect of mild and severe CIH exposure on weight gain and retroperitoneal fat in control and HF animals, respectively. The final weight of each group is also described in Table 1. A decrease in weight gain has been associated with CIH [12]. Herein, we observed that both mild and severe CIH decreased weight gain by 64 and 57% in control animals, respectively (Figure 2A). As expected, HF diet increased weight gain by 66% [33], an effect decreased in HF animals exposed to 14 days of severe CIH by 28% (Δ weight gain HF = 2.89 ± 0.22 g/day; Δ weight gain HFIH = 2.09 ± 0.59 g/day) (Figure 2A). The decrease in weight gain with CIH was not associated with a decrease in the retroperitoneal fat, since exposure to mild and severe CIH did not change the amount of this fat depot in control and HF animals (Figure 2B). Moreover, both mild and severe CIH protocols did not modify plasma NEFA in control and HF animals (Figure 2C). Adipose tissue dysfunction is usually associated with whole-body insulin resistance [34] and it has been postulated that it might contribute to metabolic dysfunction observed in OSA patients, which prompted us to evaluate adipocytes perimeter. Severe CIH exposure did not change the adipocytes perimeter, but as expected 12 weeks of HF diet intake increase the adipocytes perimeter by 40%. Interestingly, HF animals exposed to 14 days of severe CIH exhibited a decrease of 21% in adipocytes perimeter (Figure 2D).

### 3.3. Effect of Severe CIH on Adipose Tissue Hypoxia and Angiogenesis

**Figure 3:**
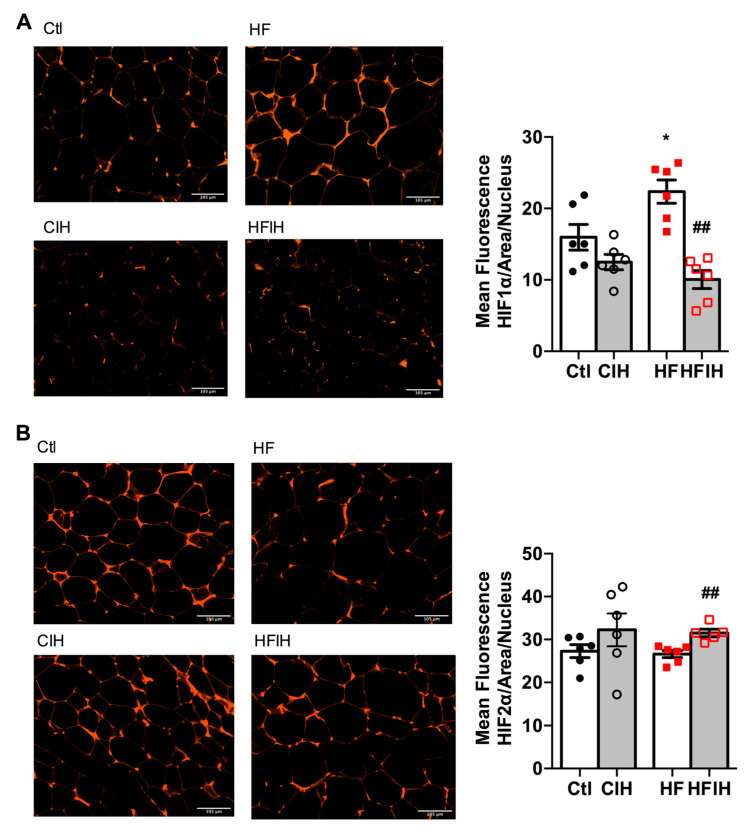
Effect of chronic intermittent hypoxia (CIH/IH) exposure on adipose tissue hypoxia in control and high-fat (HF) animals. (**A**,**B**) Immunofluorescence images for the hypoxia-inducible factor 1- and 2-alpha (HIF-1α and HIF-2α) immuno-positive staining, respectively, on adipose tissue (**right** panels) and the mean fluorescence of HIF-1α and HIF-2α, normalized for the number of nucleus (**left** panels) present in the adipose tissue of control and HF animals. Data are presented as means ± SEM of 5–6 animals. One- and two-way ANOVA with Dunnett’s and Bonferroni multiple comparison tests, respectively: * *p* < 0.05 compared with control animals; ^##^ *p* < 0.05 compared values with HF animals without CIH.

Figure 3A,B shows the effect of severe CIH on control and HF animals on hypoxia markers HIF-1ɑ and HIF-2ɑ, respectively. The right panels in Figure 3 show representative immunohistochemistry images for each group of animals and the left panels show the correspondent quantification of mean fluorescence intensity/area normalized for the number of nucleus. CIH, per se, did not modify HIF-1ɑ and HIF-2ɑ expression (Figure 3A,B). In contrast, HF diet for 12 weeks increased HIF-1ɑ in the adipose tissue by 40%, an effect completely reversed by severe CIH treatment for 2 weeks (Figure 3A). HIF-2ɑ expression in adipose tissue was not altered by HF diet consumption but severe CIH treatment in HF animals promoted an 18% increase in HIF-2ɑ (Figure 3B).

**Figure 4:**
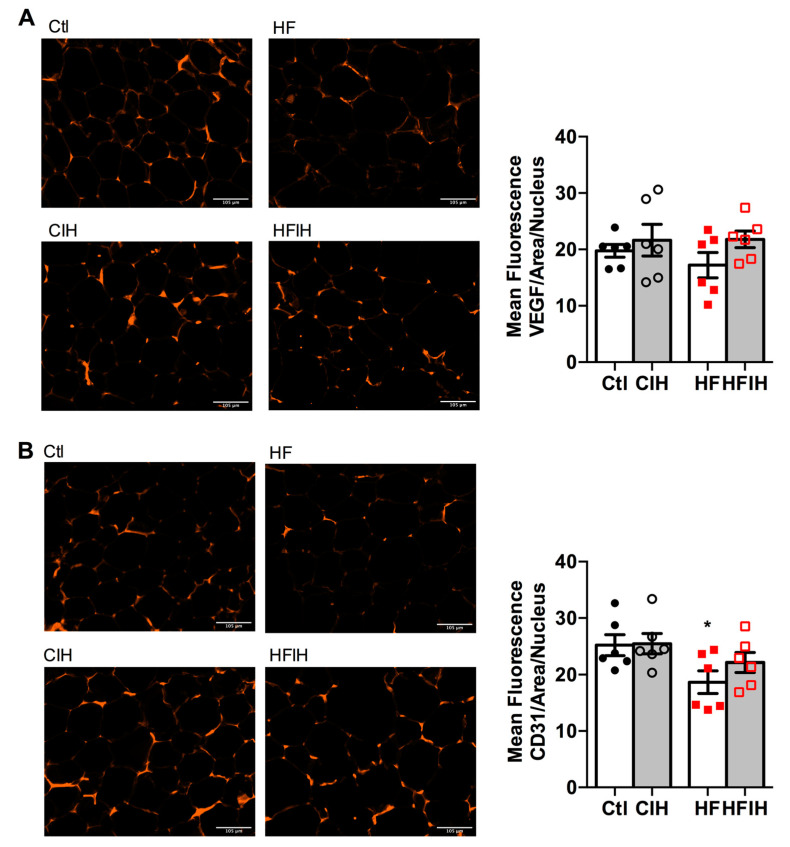
Effect of chronic intermittent hypoxia (CIH/IH) exposure on adipose tissue angiogenesis in control and high-fat (HF) animals. (**A**,**B**) Immunofluorescence images for the vascular endothelial growth factor (VEGF) and cluster of differentiation 31 (CD31) staining, respectively, on adipose tissue (right panel) and the mean fluorescence of VEGF and CD31, areas per nucleus (left panel) present in the adipose tissue of control and HF animals. Data are presented as means ± SEM of six animals. One- and two-way ANOVA with Dunnett’s and Bonferroni multiple comparison tests, respectively: * *p* < 0.05 compared with control animals.

Hypoxia is commonly associated with angiogenic processes aiming at the normalization of O_2_ levels within the tissues. Therefore, we also evaluated two angiogenesis markers, VEGF and cluster of differentiation 31 (CD31 or platelet endothelial cell adhesion molecule), in adipose tissue of control and HF animals submitted to severe CIH treatment. Figure 4A,B shows representative images for VEGF and CD31-immuno-positive staining (right panels) and the correspondent quantification of the mean fluorescence intensity in adipose tissue, respectively. Severe CIH did not alter angiogenic markers expression in the adipose tissue (Figure 4A,B). In contrast, HF diet prompted a non-significant decrease in VEGF presence (Figure 4A), while significantly decreased the expression of CD31 by 26% in comparison with control animals (Figure 4B). The CIH treatment in HF animals attenuated, in a non-significant manner, the decreased VEGF and CD31 expression in the obese model (Figure 4B).

### 3.4. Effect of Severe CIH on Adipose Tissue Hypoxia and Angiogenesis

**Figure 5:**
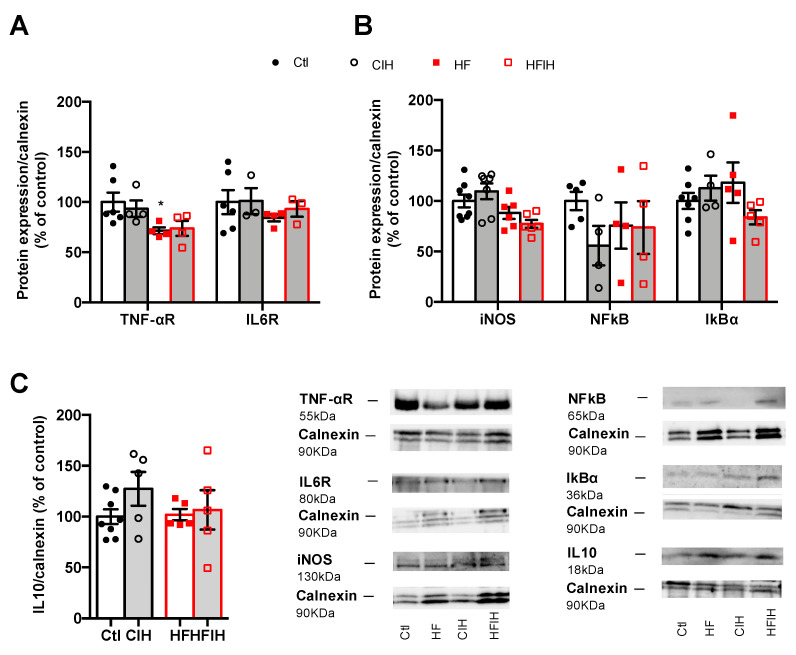
Effect of chronic intermittent hypoxia (CIH/IH) on adipose tissue inflammation in control and high-fat (HF) animals. (**A**) Effect of severe CIH on interleukin 6 receptor (IL6R, 80 kDa) and tumor necrosis factor alfa receptor (TNF-αR, 55 kDa) immunoreactivity in control and HF animals in relation to the expression of the loading protein, calnexin (90 kDa). (**B**) Effect of severe CIH exposure on inducible nitric oxide synthase (iNOS, 130 kDa), nuclear factor k B (NFkB, 65 kDa), and light polypeptide of nuclear factor k B ɑ (IkBɑ, 36 kDa) immunoreactivity in control and HF animals in relation to the expression of the loading protein, calnexin (90 kDa). (**C**) Effect of severe CIH on interleukin 10 (IL10, 18 kDa) immunoreactivity in control and HF animals in relation to the expression of the loading protein, calnexin (90 kDa). Representative Western blots for each protein studied are depicted in the right panel below. Data are presented as means ± SEM of 4–8 animals. One- and two-way ANOVA with Dunnett’s and Bonferroni multiple comparison tests, respectively: * *p* <0.05, compared with control animals.

**Figure A1:**
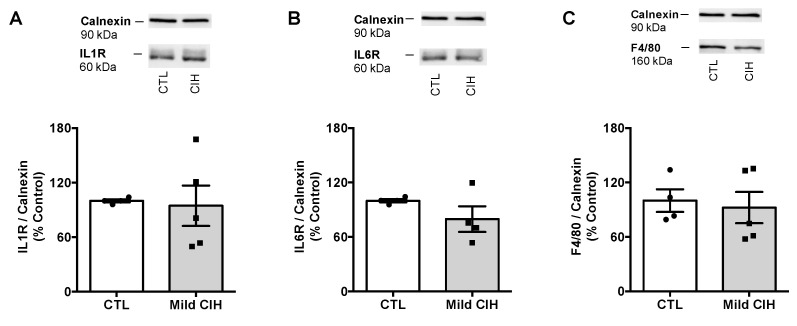
Effect of mild chronic intermittent hypoxia (CIH) exposure during 35 days on the expression of proteins involved in inflammation in the adipose tissue. Average relative interleukin 1 receptor (IL1R) (60 kDa) (**A**), interleukin 6 receptor (IL6R) (60 kDa) (**B**), and F4/80 (160 kDa) (**C**), a macrophage marker, immunoreactivity in the adipose tissue of controls and animals exposed to mild CIH expressed in relation to calnexin (90 kDa) (*n* = 4–5). Representative Western blots for each protein studied, compared to the expression in control conditions and after mild CIH exposure, are depicted on top of the respective bar graph. Data are presented as means ± SEM.

Adipose tissue inflammation has been proposed as the major mechanism behind metabolic dysfunction in OSA [18,24,25,26]. Therefore, we evaluated the effect of CIH on the expression of some inflammatory markers within the adipose tissue. Mild CIH did not alter the expression of IL1R, IL6R, and F4/80 a macrophage marker in visceral adipose tissue (Appendix A Figure A1). Moreover, severe CIH exposure for 2 weeks did not modify the expression of IL6 and TNF-α receptor and the expression of IL10 in control animals (Figure 5A,C). In HF animals, the expression of IL6 receptor and IL10 did not change with the HF diet consumption (Figure 5A,C), while the expression of TNF-α receptor decreased by 29%, an effect that is not modified when the animals were exposed for 2 weeks to severe hypoxia (Figure 5A). Additionally, severe CIH did not change the expression of iNOS, NFkB, and IkBα in the adipose tissue of control and HF animals (Figure 5B).

### 3.5. Effect of Severe CIH on Adipose Tissue Oxidative Stress

**Figure 6:**
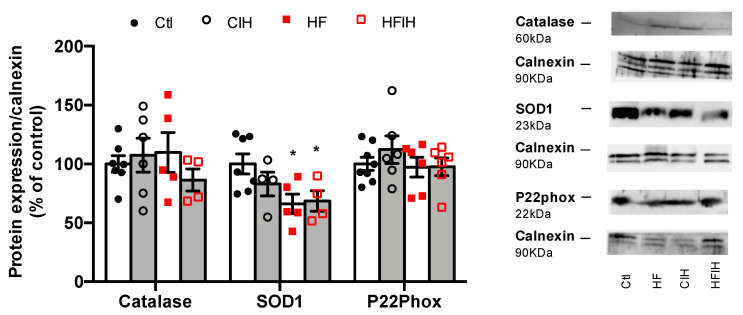
Effect of chronic intermittent hypoxia (CIH/IH) exposure on adipose tissue oxidative stress in control and high-fat (HF) animals. Effect of severe CIH on catalase (60 kDa), superoxide dismutase (SOD1, 23 kDa), and P22Phox (22 kDa) immunoreactivity in control and HF animals in relation to the expression of the loading protein, calnexin (90 kDa). Representative Western blots for each protein studied are depicted in the right panel. Data are presented as means ± SEM of 4–8 animals. One- and two-way ANOVA with Dunnett’s and Bonferroni multiple comparison tests, respectively: * *p* < 0.05, compared with control animals.

To evaluate if the oxidative stress within the adipose tissue could contribute to the early dysmetabolism observed in OSA, the expression of catalase, SOD1, and P22phox was analyzed (Figure 6). Severe CIH did not modify the expression of catalase and P22phox in both control and HF animals. The expression of SOD1 was not modified in control animals exposed to severe CIH, but an HF diet decreased SOD1 expression by 34%, an effect not modified when HF animals were exposed to 14 days of severe CIH (Figure 6).

### 3.6. Effect of Severe CIH on Adipose Tissue Metabolism

**Figure 7:**
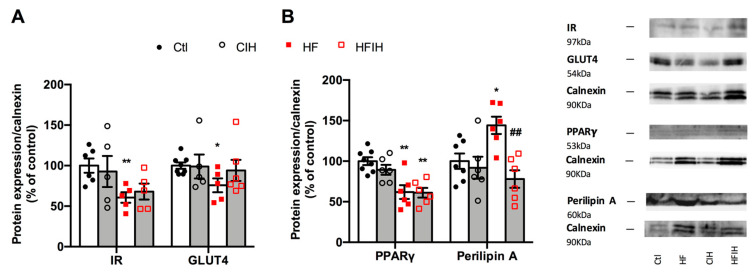
Effect of severe chronic intermittent hypoxia (CIH/IH) exposure on the metabolism of the adipose tissue. (**A**) Effect of severe chronic intermittent hypoxia (CIH/IH) exposure on adipose tissue (97 kDa) and glucose transporter GLUT4 (54 kDa) immunoreactivity in control and HF animals in relation to the expression of the loading protein, calnexin. (**B**) Effect of severe CIH on peroxisome proliferator-activated receptor γ (PPARγ) (53 kDa) and perilipin A (60 kDa) immunoreactivity in control and HF animals in relation to the expression of the loading protein, calnexin (90 kDa). Representative Western blots for each protein studied are depicted in the right panel. Data are presented as means ± SEM of 5–8 animals. One- and two-way ANOVA with Dunnett’s and Bonferroni multiple comparison tests, respectively: * *p* < 0.05 and ** *p* < 0.01, compared with control animals; ^##^ *p* < 0.05 compared values with HF animals without CIH.

Figure 7 shows the effect of severe CIH exposure on the metabolism of adipose tissue in control and HF animals. Severe CIH, per se, did not modify the expression of the insulin receptor and GLUT4. As expected, the HF diet decreased insulin receptor and GLUT4 expression [33] by 39 and 24%, respectively (Figure 7A). Severe CIH did not modify the expression of the insulin receptor in the HF animals, while increases non-significantly by 24% the expression of GLUT4. The expression of PPARγ, a nuclear receptor and transcription factor that regulates adipocyte differentiation, and perilipin A, a protein involved in lipid storage and fatty acid release, were also evaluated [34]. Severe CIH, per se, did not alter PPARγ and perilipin A expression within the adipose tissue (Figure 7B). In contrast, the HF diet decreased adipose tissue levels of PPARγ by 38% and increased the expression of perilipin A by 44%. CIH in HF animals did not modify PPARγ levels but decreased perilipin A by 85% (Figure 7B).

## 4. Discussion

**Figure 8:**
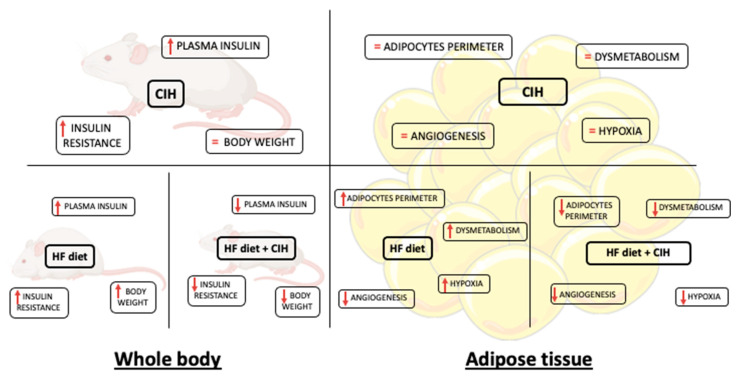
Schematic figure representing the major findings of the effect of chronic intermittent hypoxia (30 hypoxic (5% O_2_) cycles, 8 h/day) for 15 days, on body weight and whole-body insulin sensitivity and levels and adipose tissue function in control and obese rats.

In the present study, we demonstrated that CIH-induced early-stage metabolic dysfunction, characterized by hyperinsulinemia and whole-body insulin resistance, without alterations in weight gain, in adipocytes perimeter, and in adipose tissue hypoxia, angiogenesis, oxidative stress, and metabolism. Moreover, and surprisingly, severe CIH exposure during 14 days in HF animals attenuated the dysfunction of adipose tissue induced by the HF diet (Figure 8).

As expected, and previously described, exposure to mild CIH or severe CIH-induced whole-body insulin resistance and increased fasting insulin levels [11,12,25,29,35,36]. In fact, the exact same paradigms of CIH were already tested in rats originating the same degree of whole-body metabolic dysfunction showed herein [11,12]. Moreover, and as previously described [25], obesity-induced by the intake of hypercaloric diets—that is known to be a risk factor for the development of OSA, while prompted severe insulin resistance and hyperinsulinemia, in the presence of CIH did not aggravate metabolic dysfunction (Table 1). Several evidences reported that OSA leads to the development of metabolic dysfunction and aggravates obesity [3,4]. However, herein the CIH-induced metabolic dysfunction was associated with decreased weight gain. This is in agreement with the presence of pathological metabolic features in lean OSA patients [8,9], and with the previous results showing that exposure of mice to CIH decreases weight gain [10,13,14,35]. Interestingly, CIH decreases weight gain not only in control animals but also in HF animals, this effect not being due to a decrease in retroperitoneal fat (Figure 2A,B). One could postulate several mechanisms behind this effect, as a loss of muscle mass. However, the reduction in adipocytes perimeter in the HF animals when exposed to CIH in comparison with the HF rats, suggests that a higher metabolism of the adipose tissue and/or increased energy expenditure might be responsible for the reduction in weight [37]. However, this clearly contrasts with the recent hypothesis that adipose tissue dysfunction and particularly adipose tissue hypoxia and inflammation are key pathophysiological mechanisms responsible for metabolic dysfunction in CIH and therefore in OSA [25,27,29,35].

Hypoxia and consequently angiogenesis are main factors for adipose tissue dysfunction particularly in obesity [38,39]. Moreover, Gozal et al. found that CIH (20 cycles/h 6 h) during 6 weeks in mice decreased angiogenesis measured through the mRNA expression of VEGF and immunohistochemical evaluation of CD31 with no alterations in adipose tissue HIF-1α expression [29]. The absence of results in adipose tissue hypoxia with CIH is in agreement with lack of alterations in HIF-1α or HIF-2α in the adipose tissue in rats exposed to mild CIH [12] and with the results herein obtained that severe CIH during 2 weeks did not modify HIF-1α or HIF-2α. As expected, obesity increased significantly HIF-1α expression in visceral adipose tissue, an effect that was reversed by the exposure of obese animals to CIH. In accordance, severe CIH during 2 weeks did not change angiogenesis markers in control animals, while restored the immuno-positive staining for VEGF and CD31 in HF animals. Altogether, these results agree with the decrease in adipocytes perimeter found in obese animals exposed to CIH and suggest a protective role for CIH in some factors that usually contribute to adipose tissue dysfunction in obesity as postulated by some authors [40].

Another major factor that contributes to adipose tissue dysfunction is inflammation. It has been shown that adipose tissue in mice exposed to 6 [25] or 20 weeks of CIH [35] and cultured CIH adipocytes exhibit an inflammatory phenotype independently of obesity, which originated the hypothesis that inflammation of visceral adipose tissue was the main cause of metabolic dysfunction seen in OSA patients. However, herein we show that neither mild CIH (Appendix A Figure A1) nor 2 weeks of severe CIH (Figure 5) produced a pro-inflammatory profile within the visceral adipose tissue assessed by measuring the levels of expression of the receptors of pro-inflammatory molecules—TNF-α, IL1, and IL6, through the expression of enzymes—iNOS and transcription factors—NFkB, IkBɑ involved in inflammatory pathways. These results are in the same line of the findings by Poulain et al. where they found that TNF-α and IL6 secretion from the adipose tissue in mice were not affected by CIH [36]. Moreover, interestingly was the fact that severe CIH during 2 weeks showed a tendency to increase IL-10, an anti-inflammatory interleukin, which might represent compensatory mechanisms to counteract the development of inflammation of the adipose tissue in CIH. Obesity as expected produced a pro-inflammatory phenotype in the visceral adipose tissue [39,41], evidenced by a significant decrease in the TNF-αR and a tendency to decrease in IL6R, suggesting that increased levels of these pro-inflammatory mediators are probably associated with a decreased insulin sensitivity. Herein, we also show that CIH exposure in obese rats did not change the inflammatory status, this being in agreement with the findings of Perrini et al., where they found that CIH does not represent an additional factor for increasing systemic and adipose tissue inflammation in morbid obesity [42].

Oxidative stress is another major factor of adipose tissue dysfunction as it can cause an increase in preadipocyte proliferation, adipocyte differentiation, and the size of mature adipocytes [43]. Additionally, oxidative stress induces insulin resistance of adipocytes and increases secretion of several cytokines and pro-inflammatory interleukins, IL-6 and TNF-α by adipocytes [44]. Herein, we showed that severe CIH during 2 weeks does not seem to change the anti-oxidant defenses of the adipose tissue, measured as catalase, SOD1, and p22Phox—a critical component of the superoxide-generating NADH/NADPH oxidase system. In contrast, and as expected, obesity produced a significant decrease in the front line of defense against reactive oxygen species—SOD1 expression—that is not modified by CIH. The absence of effects of CIH on the anti-oxidant defenses contrasts with the findings by Gileles-Hillel et al. that described an increase in ROS in visceral adipose tissue infiltrated macrophages [35]. However, our results showing absence of CIH effect on ROS might be due to the lack of inflammation seen in the adipose tissue in our CIH paradigms.

In agreement with the lack of effects of CIH on hypoxia, inflammation, angiogenesis, and oxidative stress in our 2 weeks severe CIH paradigm, we did not observe any alterations in adipose tissue insulin resistance, as insulin receptor expression and glut4 transporter did not change. Accordingly, PPARγ, a master regulator of adipogenesis in mammals causing insulin sensitization and enhancing glucose metabolism [45] did not change with CIH. Obesity, as expected, induced insulin resistance in the adipose tissue, seen by a decreased insulin receptor, Glut4 transporter, and PPARγ levels within the visceral adipose tissue, effects not modified when obese animals were exposed to CIH. This absence of adipose tissue insulin resistance and dysfunction in CIH together with the development of whole-body insulin resistance clearly means that adipose tissue dysfunction is not the initial event that triggers metabolic disease in OSA.

Moreover, and trying to find a mechanistic answer to the effect of CIH in adipocytes perimeter, we evaluated perilipin A, a key regulator of lipolysis and we found that although CIH did not modify its expression in control animals it completely reversed the increase in its expression produced by the HF diet, suggesting that 2 weeks of CIH in an initial stage of metabolic dysfunction promotes lipolysis, therefore having a protective role in the dysfunction of the adipose tissue. However, we cannot rule out a possible role of adipose tissue dysfunction in maintaining dysmetabolic states in more severe and longer stages of CIH/OSA. In fact, some of the huge drawbacks to make clear conclusions on the role of CIH in metabolic diseases development among all the existing literature are the different times of exposure and the different paradigms of disease studied, as well as the different species evaluated. For example, it has been shown that short exposures to CIH may have protective effects on the damage related to exposure to severe hypoxia in contrast with the deleterious effects of long-term CIH exposure (for a review, see [40,46]). In the same line, different species and strains of animals might produce contradictory results, as seen by Ge et al. where two different substrains of lean and obese C57BL/6 mice exhibited differential metabolic and inflammatory responses to intermittent hypoxia [47].

## 5. Conclusions

In conclusion, our results demonstrate for the first time that CIH induce early metabolic dysfunction independently of adipose tissue hypoxia, angiogenesis, inflammation, and oxidative stress. Moreover, we found that short-term exposure to CIH in obesity might reverse/protect against the deleterious effects of obesity in adipose tissue function.

## Figures and Tables

**Table 1:** Effect of mild and severe chronic intermittent hypoxia (CIH) exposure on weight, fasting glycemia, fasting insulinemia, and insulin resistance calculated by the homeostatic model assessment (HOMA-IR) method in control and high-fat (HF) animals.

	Mild		Severe			
	Control	CIH	Control	CIH	HF	HFIH
Weight (g)	382 ± 28	355 ± 8	492 ± 9	457 ± 10 *	696 ± 7	609 ± 38 *
Glycemia (mmol/L)	5.12 ± 0.20	4.78 ± 0.26	4.84 ± 0.13	4.63 ± 0.14	4.99 ± 0.13	4.76 ± 0.10
Insulin (pmol/L)	231.40 ± 31.23	529.70 ± 9.65 ****	300.70 ± 61.53	570.10 ± 61.53 *	657.50 ± 104.70 *	631.90 ± 96.89
HOMA-IR	5.22 ± 0.65	12.00 ± 0.84 ****	6.18 ± 0.91	12.02 ± 1.64 **	15.28 ± 1.45 **	14.22 ± 2.37

Data are means ± SEM of six to eight rats. Two-way ANOVA with Bonferroni multiple comparison test: * *p* < 0.05, ** *p* < 0.01, and **** *p* < 0.0001 compared with control animals of the respective groups.

## Data Availability

The data are not publicly available due to a lack of time between data obtention and publication. The data that support the findings of this study are available from the corresponding author upon request.
